# Uncovering the pattern of physical behaviours among Swedish ambulance personnel: is the type of work shift important?

**DOI:** 10.1186/s12889-026-27335-y

**Published:** 2026-04-21

**Authors:** Anna M. Johnsen, Nidhi Gupta, Stavros Kyriakidis, Anders Broström, Petra Wagman, Eleonor I. Fransson

**Affiliations:** 1 Department of Nursing, School of Health and Welfare, Jönköping University, Jönköping, Sweden; 2 Department of Musculoskeletal Disorders and Physical Work Demands, National Research Centre for the Working Environment, Copenhagen, Denmark; 3 Department of Sports Science and Clinical Biomechanics, Faculty of Health Sciences, Research Group of Physical Activity and Health in Working Life, Southern Denmark University, Copenhagen, Denmark; 4 Department of Clinical Neurophysiology, Linköping University Hospital, Linköping, Sweden; 5 Department of Rehabilitation, School of Health and Welfare, Jönköping University, Jönköping, Sweden; 6 School of Health and Welfare, Jönköping University, Jönköping, Sweden

**Keywords:** Ambulance personnel, Physical behaviour, Shift work, Accelerometer, Work environment, Leisure, Compositional data analysis

## Abstract

**Background:**

Previous studies indicate that ambulance personnel have an increased risk of ill health. Shift work and time spent on physical behaviours during work and leisure are factors that could be related to health, however the research is limited. Thus, the aim of this study was to describe patterns of physical behaviours during and after work among Swedish ambulance personnel and to analyse the associations between physical behaviours and different work shifts.

**Methods:**

In this observational study, the physical behaviours of 63 ambulance personnel were measured over seven days using two accelerometers. Accelerometer data was processed using the MATLAB program Acti4, to identify physical behaviours i.e. sleep, being sedentary, light physical activity (LPA), and moderate to vigorous physical activity (MVPA), during and after work. To determine the association between shift types (independent) and patterns of physical behaviours (dependent), a Multivariate Analysis of Variance was performed on data processed according to compositional data analysis.

**Results:**

At work, the highest proportion of both MVPA and being sedentary occurred during day shifts, compared to night and 24-h shifts (MVPA: 7% vs 4% and 5%; sedentary time: 62% vs 44% and 54% respectively). Night and 24-h shifts included 31% and 18% sleep, respectively. During the after-work periods, the highest proportions of MVPA were observed after 24-h shifts (8%). Overall, there was no statistically significant difference in physical behaviours during work and after work for various shift types. However, in a sub-analysis restricted to night and 24-h shifts, a statistically significant association between shift type and composition of physical behaviours during work was observed (η_p_^2^ = 0.42, *p <* 0.001), with a lower proportion of sleep time relative to awake time during 24-h shifts compared to night shift (η_p_^2^ = 0.34, *p <* 0.001).

**Conclusions:**

In general, ambulance personnel were physically active both during and after work. At the same time, work hours entailed a substantial amount of sedentary time. Shift type was not associated with the pattern of physical behaviours among ambulance personnel. However, during 24-h shift a lower proportion of the time was spent sleeping compared to during night shift. Studies with larger sample sizes are needed to confirm these results.

**Supplementary Information:**

The online version contains supplementary material available at 10.1186/s12889-026-27335-y.

## Background

Ambulance personnel in the emergency medical services (EMS) provide advanced pre-hospital care to individuals in need. They are thereby a very important group in society, and their competence is needed to be able to maintain a chain of high-quality care to every patient, regardless of location [1]. Thus, maintaining and improving the health of the ambulance personnel is essential.

Findings from previous research have indicated an increased prevalence of ill health among ambulance personnel compared to other occupational groups, e.g., increased risk for cardiovascular diseases [2, 3] and musculoskeletal disorders [2, 4, 5]. Despite this knowledge, very limited research on maintaining and improving health has been done on ambulance personnel. Thus, it is essential to explore how various factors influence the health of this group.

Available research has shown that the work environment within the EMS includes a number of risk factors for ill health, such as stress [6], long working hours, and heavy lifting [7]. Another risk factor of importance could be the pattern of physical behaviours, i.e. physical activities, sedentary behaviour, and sleep, during work [8]. The work in the EMS includes physical activity due to the physical demands, but it also includes periods of sedentary time, such as sitting in the ambulances or waiting at the station between emergency calls [8]. During recent decades, the number of emergency calls has increased [9], which might negatively affect the possibility of recovery between calls. Moreover, this may limit the opportunities to rest after physically demanding tasks and limit the time available for exercise at the workplace gym. Furthermore, it is possible that the pattern of physical behaviours during work could affect the ambulance personnel’s energy to exercise or to perform other beneficial physical activities after work. The benefits of physical activity on health are widely known [10]. However, conflicting evidence exists regarding whether physical activity during work i.e. occupational physical activity, has the same positive effect as leisure-time physical activity [11]. To the best of our knowledge, the pattern of physical behaviours has not been investigated among ambulance personnel separately for work and after work.

Shift work is another factor that might affect the health of ambulance personnel [7, 12]. Besides the associations between shift work and various health outcomes [13], shift work may also affect the pattern of physical behaviours both during work and after work for this group [14]. However, such an association between shift work and patterns of physical behaviours has not previously been explored among ambulance personnel. Therefore, the aim of this study was to describe the patterns of physical behaviours during working days, both during and after work, among Swedish ambulance personnel. A further aim was to analyse the associations between physical behaviours and different work shifts.

## Method

### Study design and setting

This study used a quantitative, observational design with objective measurements of physical behaviour during and after work among ambulance personnel. The data collection was conducted between May 2021 and June 2022 in an EMS in the south of Sweden. The EMS included 10 ambulance stations and was divided into three subdivisions based on geographic location. The three subdivisions had small differences regarding the organisation of schedules and shift lengths. However, all divisions had day shifts (most commonly ranging from 07:00–17:00), night shifts (most commonly ranging from 17:00–08:00), and 24-h shifts (most commonly from 07:00–07:00). The schedule for most of the ambulance personnel was a mix of all three shift types, and it was common to have all three different shift types during the same week. The ambulance personnel were allowed to sleep during night shifts and 24-h shifts if no emergency calls were assigned to the EMS. Moreover, they had access to exercise facilities and were allowed to exercise during work time between emergency calls.

### Participants and data collection

In 2021, approximately 190 employees were working in the EMS. Of these, about 85% were registered nurses and about 15% were emergency medical technicians. All permanently employed registered nurses and emergency medical technicians engaged in work including patient care (*n =* 184), were invited to participate in this study and 66 agreed to participate. During the data collection period, three participants announced that they were no longer available for participation in the study, resulting in 63 participants in the study. The data collection consisted of physical behaviours measured over seven days and nights using triaxial accelerometers, including a daily short diary, and a questionnaire. More details regarding the procedure during the data collection are available in additional file 1.

### Assessment of physical behaviour

Triaxial accelerometers (Axivity AX3, Axivity Ltd, Newcastle Upon Tyne, UK), were used to objectively measure physical behaviours during work and non-work time. The manufacturer’s software (OMGUI Version 1.0.0.43; Axivity Ltd, Newcastle upon Tyne, UK) was used for initialisation of the accelerometers and for downloading data after the measurement. A sampling rate of 25 Hz was used. The two accelerometers were synchronised before and after the measurement period to avoid discrepancies in recording time.

**Fig. 1:**
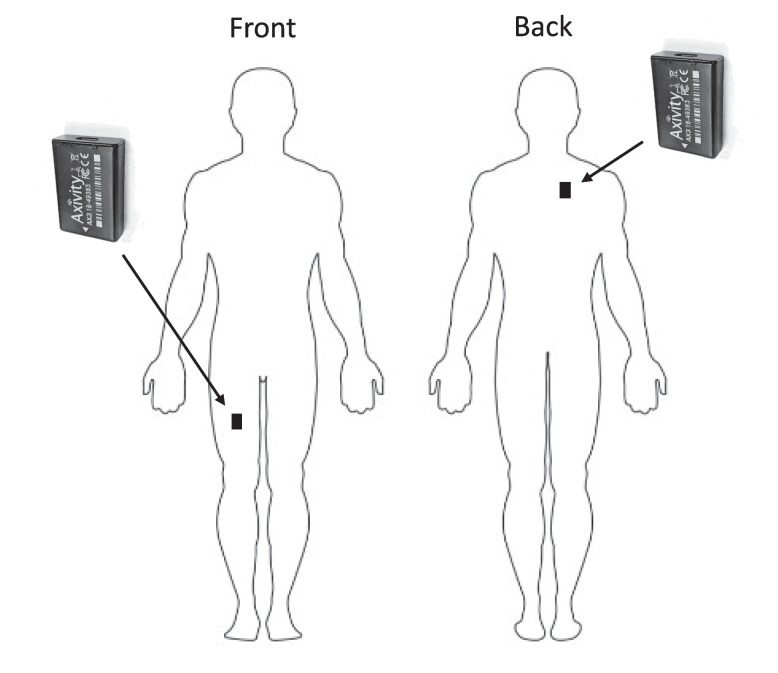
The accelerometers attached to the thigh and upper back

The accelerometers were attached by adhesive tape (German Brown, Walker Tape®) and covered with plastic film (Opsite Flexifix, Smith & nephew®). One accelerometer was attached on the front of the right thigh, midway between the hip and the knee joint, and one on the upper back close to the spine at the level of T1/T2 (Fig. 1).

The MATLAB program Acti4 (National Research Centre for the Working Environment, Copenhagen, Denmark) was used for further analysis. The Acti4 program has previously shown high validity in detecting physical behaviours e.g. sitting, standing, moving, walking, running, walking stairs, cycling, and rowing during work and leisure [15, 16]. The identification of postures (lying, sitting, standing and moving) was based on the inclination of the thigh and the trunk. The identification of the movements (i.e. stair climbing, walking fast and slow, running and cycling) was based on various cut-off points of raw acceleration (g) [15]. Acti4 software does not use the traditional epoch length to classify raw acceleration data into postures and movements. Instead, it classifies all raw acceleration data in 2-s intervals with 50% overlap into postures and movements [17]. The combination of one accelerometer on the thigh and one on the trunk, enabled to also differentiate between sitting and lying [15]. The daily short diary was used to classify the accelerometer registrations as sleep, work, or after work, see additional file for more information.

Physical behaviours were classified into four categories: 1) sleep (information from the diary, combined with registrations from the accelerometers); 2) being sedentary (lying and sitting, registered by the accelerometers); 3) light physical activity (LPA) (standing, moving, and walking slowly < 100 steps/min, registered by the accelerometers); 4) moderate to vigorous physical activity (MVPA) (walking fast > 100 steps/min, running, climbing stairs, cycling, and rowing, registered by the accelerometers). Together with the information from the diary, the four categories of physical behaviours could be specified for work and after work. More details regarding the daily diary are provided in additional file 1.

**Fig. 2:**
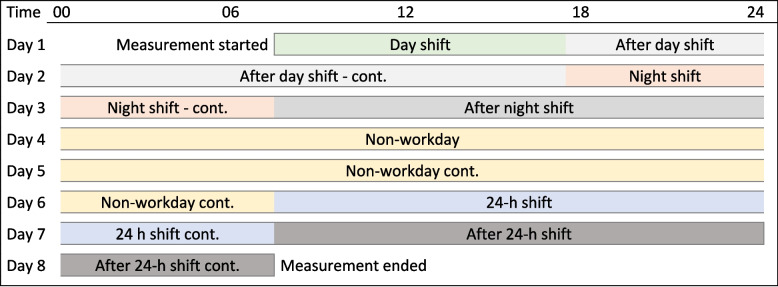
An example of categorisation of time into seven different categories during the measurement period

Using the information entered in the daily diary, the physical behaviours during seven days could be grouped in seven different categories: 1) day shift, 2) night shift, 3) 24-h shift, 4) after day shift, 5) after night shift, 6) after 24-h shift, and 7) non-workday, based on the participants’ notes (Fig. 2). A non-workday was defined as no working hours from midnight to midnight, continuing until the next work shift started.

### Questionnaire

A web-based questionnaire was sent to the participant’s e-mail no more than three weeks after the measurement was completed. The questionnaire comprised questions regarding background characteristics of age, sex, occupation, and years working in the EMS. The questionnaire also covered items on health, working conditions and lifestyle, including: the Work Ability Index (WAI) [18], self-rated health, self-reported sleep, self-reported leisure-time physical activity, and self-reported weight and height which were used to calculate body mass index (BMI). Detailed information regarding the questionnaire, including scales, ranges, and categorisation, is provided in additional file 1.

### Statistical analysis

#### Descriptive analysis

**Fig. 3:**
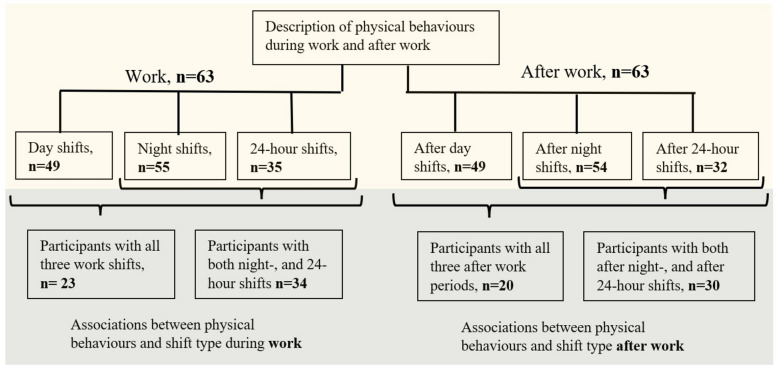
Overview of the number of participants in the different analyses and sub-samples

Since the aim of this study was to analyse working days, only working days were included (i.e. omitting the category 7 “non-workday”). A work or after-work period of less than six hours was omitted from the analysis, as these were considered atypical periods, resulting in four recorded time periods being excluded. For the whole sample (*n =* 63), the mean time (hours) in sleep, being sedentary, in LPA, and in MVPA during the six included time categories was calculated and averaged for categories of the same type, for each participant. Since not all participants had all types of shifts during the seven-day monitoring period, the number of participants contributing data differed across shifts (49 day-, 55 night-, and 35 24-h shifts) and across the corresponding after-work periods (49 after day, 54 after night, and 32 after 24-h shifts; see Fig. 3). The results are presented as mean hours, with standard deviation, and range. Moreover, the proportions of sleep, being sedentary, LPA, and MVPA during the different time categories were calculated. Background characteristics, health, work conditions, and lifestyle are presented by total numbers and proportions, or means and standard deviations. Ternary plots are used to illustrate the variation in time being sedentary, LPA, and MVPA during work and after-work hours. To enable comparison of the results from this study with other samples where physical behaviour is measured over 24 h from midnight to midnight, average hours of device-based physical behaviours across complete working days were also calculated.

#### Compositional data analysis, physical behaviour, and shift types

In this study, the physical behaviours sleep, being sedentary, LPA, and MVPA, and their association with shift types, were analysed using compositional data analysis (CoDA) [19]. When the time is constrained to a certain period, e.g., a work shift or a period after a work shift, an increased time spent in one type of physical behaviour will lead to less time spent in other physical behaviours during that specified period. Therefore, it has been suggested that different physical behaviours should not be analysed separately, but instead should be analysed as one composition [20, 21].

The durations of the physical behaviours sleep, being sedentary, LPA and MVPA for each of the six time categories were transformed into isometric log ratios (ILRs) [22, 23]. Two different sets of ILRs were used, one including a composition of three parts (being sedentary, LPA, and MVPA) and one including a composition of four parts (sleep, being sedentary, LPA, and MVPA). Details on how the ILRs were calculated are given in additional file 1. Since the time categories of interest were the different work shifts and their corresponding time categories after work, the analysis was based on the composition of the activities during these time categories, unlike previous studies that used the composition of a “24-h time” period. Any non-wear time, when participants removed the accelerometers, was excluded from the data without imputation.

As the ambulance personnel were allowed to sleep when possible, during night shifts and 24-h shifts, time spent sleeping was recorded during these shifts. However, during day shifts, no sleep was present. Zero sleep during day shifts was therefore considered as essential zeros [24] in this study. Since CoDA is not robust at handling essential zeros, when analysing the effects of all three shifts, we decided to remove the sleep from all compositions (see additional file 1 for how we defined ILRs for such comparison). In sub-analyses based on night shifts and 24-h shifts, and their corresponding after-work periods, we also included sleep in the composition. During night shifts and 24-h shifts six observations had zero sleep. During the corresponding after-work periods, six observations had zero sleep. The zero sleep during these periods was considered as rounded zeros, and therefore an imputation was performed using the log-ratio Expectation–Maximisation method [25].

To analyse physical behaviour in relation to shift types, a repeated-measures multivariate analysis of variance was applied, with shift type as a within-subject factor, and the ILRs as dependent variables. In total, 23 participants had registrations from all three shift types and 20 participants had registrations from all the corresponding after-work periods. In the sub-analysis based on participants with night shifts and 24-h shifts, 34 participants were included, and 30 participants had registrations from the corresponding after-work periods (Fig. 3). For the repeated-measures MANOVA, normality of the ILR-transformed dependent variables was assessed and no deviation from normality was observed. Since a repeated-measures design was used, correlations within participants across repeated measurements were accounted for by the model. Following the repeated-measures MANOVA, univariate tests were conducted for each ILR-transformed dependent variable to examine the effect of shift. Partial eta square (η_p_^2^) was used to measure the effect size, where the cut-offs 0.0099, 0.0588, and 0.1379 were used to indicate weak, moderate, and strong effects [26]. The level of statistical significance was set at 0.05. All statistical analyses were performed using IBM SPSS Statistics for Windows (version 27), and R (version 4.2.2) using the ‘compositions’ [27], ‘zCompositions’ [28], and ‘ggtern’ [29] packages.

## Results

Based on data from the 63 ambulance personnel, with a mean accelerometer wear time of 165 h (standard deviatio*n =* 19), 441 time categories referring to working days were included in the analysis. These comprised 229 during-work periods, covering all three work shifts, and 212 after-work periods, covering all three after work categories. Participants contributed, on average, to 3.6 of these included time categories (range 2–6) during work and 3.4 (range 1–6) after work. The average total work time during the included working days was 49.7 h (range 18.8—84.0), and the corresponding total time after work was 57.3 h (range 16.5—108.5). In total, 6738 h of accelerometer data were analysed.

The background characteristics of the participating ambulance personnel are presented in Table 1. The mean age was 46 years, two out of five were women, more than 90% were registered nurses, and the average work experience from EMS was 16 years. The work ability was reported as good or very good among 87% of the participants and 70% reported moderate or vigorous leisure-time physical activity based on self-report from the questionnaire. The self-rated sleep length was 6.8 h and 68% reported good or very good sleep quality.

**Table 1:** Descriptive statistics for 63 ambulance personnel

Age, mean (SD)	46 (10.4)
Sex, n (%)	
Women	24 (38)
Men	39 (62)
Occupation, n (%)	
Registered nurses	58 (92)
Emergency Medical Technicians	5 (8)
Years working in EMS, mean (SD), *n =* 61	16 (9.5)
WAI, mean (SD), *n =* 60	42.4 (4.7)
Very good	29 (48.3)
Good	23 (38.3)
Moderate	8 (13.3)
Bad	0 (0)
Self-rated health, mean (SD)	4.2 (0.8)
Self-rated overall leisure time physical activity level, n (%)	
Sedentary	0 (0)
Light	18 (30)
Moderate	19 (32)
Vigorous	23 (38)
Device based physical behaviours (hours) from midnight to midnight during complete workdays, mean (SD), percentage of 24 h	
Sleep	6.97 (0.97), 29%
Sedentary	10.30 (1.35), 43%
LPA	5.26 (1.00), 22%
MVPA	1.45 (0.48), 6%
BMI, mean (SD), *n =* 60	24.9 (3.3)
Normal	35 (58)
Overweight	21 (35)
Obese	4 (7)
Self-rated sleep length, hours, mean (SD), *n =* 59	6.8 (1.0)
Self-rated sleep quality, n (%), *n =* 60	
Bad or very bad	4 (7)
Varies	15 (25)
Good or very good	41 (68)

*EMS* Emergency Medical Services, *WAI* Work Ability Index, *LPA* Light Physical Activity, *MVPA* Moderate to vigorous physical activity, *BMI* Body Mass Index

The mean time spent in different physical activities during work, stratified by shift, is presented in Table 2. The average time spent sleeping during night shifts and 24-h shifts was quite similar (4.46 vs. 4.23 h). However, since the total work time during night shifts and 24-h shifts differed by almost 10 h, the proportion of sleep differed (31% during night shift vs. 18% during 24-h shifts). During these shifts, the total sleep time was the summary of up to five shorter sessions. No sleep was present during day shifts; however, the proportion of time being sedentary was highest during day shifts compared to night shifts and 24-h shifts. The proportion of time spent in MVPA was highest during day shifts with 7% compared to 4% during night shifts and 5% during 24-h shifts. When comparing awake time across the three work shifts, sedentary time was highest during 24-h shifts (66%), followed by night shifts (64%) and day shifts (62%).

**Table 2:** Mean time spent in different activities during three different work shifts (*n =* 63)

	Day shift (111 shifts), *n =* 49			Night shift (81 shifts), *n =* 55			24-h shift (37 shifts), *n =* 35		
	Mean h (SD)	%	Range	Mean h (SD)	%	Range	Mean h (SD)	%	Range
Total time	9.86 (1.33)		6.25–12.75	14.34 (1.15)		11.79–17.0	24.07 (0.46)		22.5–25.0
Sleep	0 (0)	0%		4.46 (2.36)	31%	0–9.5	4.23 (2.19)	18%	0–8.0
Sedentary	6.04 (1.21)	62%	2.31–8.16	6.37 (1.99)	44%	1.97–11.67	12.90 (2.05)	54%	8.82–18.41
LPA	3.13 (1.16)	31%	1.21–7.16	2.91 (1.14)	20%	0.73–6.73	5.65 (1.48)	23%	3.23–9.67
MVPA	0.70 (0.30)	7%	0.19–1.53	0.60 (0.26)	4%	0.15–1.53	1.29 (0.44)	5%	0.66–2.33

*LPA* Light physical activity, *MVPA* Moderate to vigorous physical activity, *h* hours, *SD* Standard Deviation

The corresponding mean time spent in different activities after work is presented in Table 3. The average time spent sleeping was quite similar during the three after-work periods, ranging from 5.37 after night shifts, to 6.03 h after day shifts. However, due to the different lengths of the periods, the proportion of sleep after day shifts was higher than after night shifts and 24-h shifts (41% vs 25% and 26%). The proportion of time being sedentary was lower after day shifts compared to after night shifts and 24-h shifts. The mean time spent in MVPA was 0.88 h after day shifts, 1.41 h after night shifts, and 1.58 h after 24-h shifts. When comparing awake time across the three after-work categories, sedentary time was similar across all categories, at approximately 60%.

**Table 3:** Mean time spent in different activities during three different after-work periods (*n =* 63)

	After day shift (105 periods), *n =* 49			After night shift (73 periods), *n =* 54			After 24-h shift (34 periods), *n =* 32		
	Mean h (SD)	%	Range	Mean h (SD)	%	Range	Mean h (SD)	%	Range
Total time	14.44 (5.50)		6.0–27.25	20.13 (4.63)		10.33–32.5	20.22 (7.49)		7.50–34.0
Sleep	6.03 (2.59)	41%	0.5–12.5	5.37 (3.53)	25%	0–12.17	5.62 (3.76)	26%	0.0–14.0
Sedentary	4.86 (1.76)	35%	1.73–8.70	8.67 (2.22)	45%	5.22–15.89	8.53 (2.99)	45%	3.36–17.61
LPA	2.66 (1.54)	18%	0.60–6.86	4.72 (1.58)	23%	1.29–9.02	4.49 (2.13)	22%	0.56–7.97
MVPA	0.88 (0.71)	6%	0.14–3.31	1.41 (0.77)	7%	0.25–3.37	1.58 (0.99)	8%	0.26–5.01

*LPA* Light physical activity, *MVPA* Moderate to vigorous physical activity, *h* hours, *SD* Standard Deviation

**Fig. 4:**
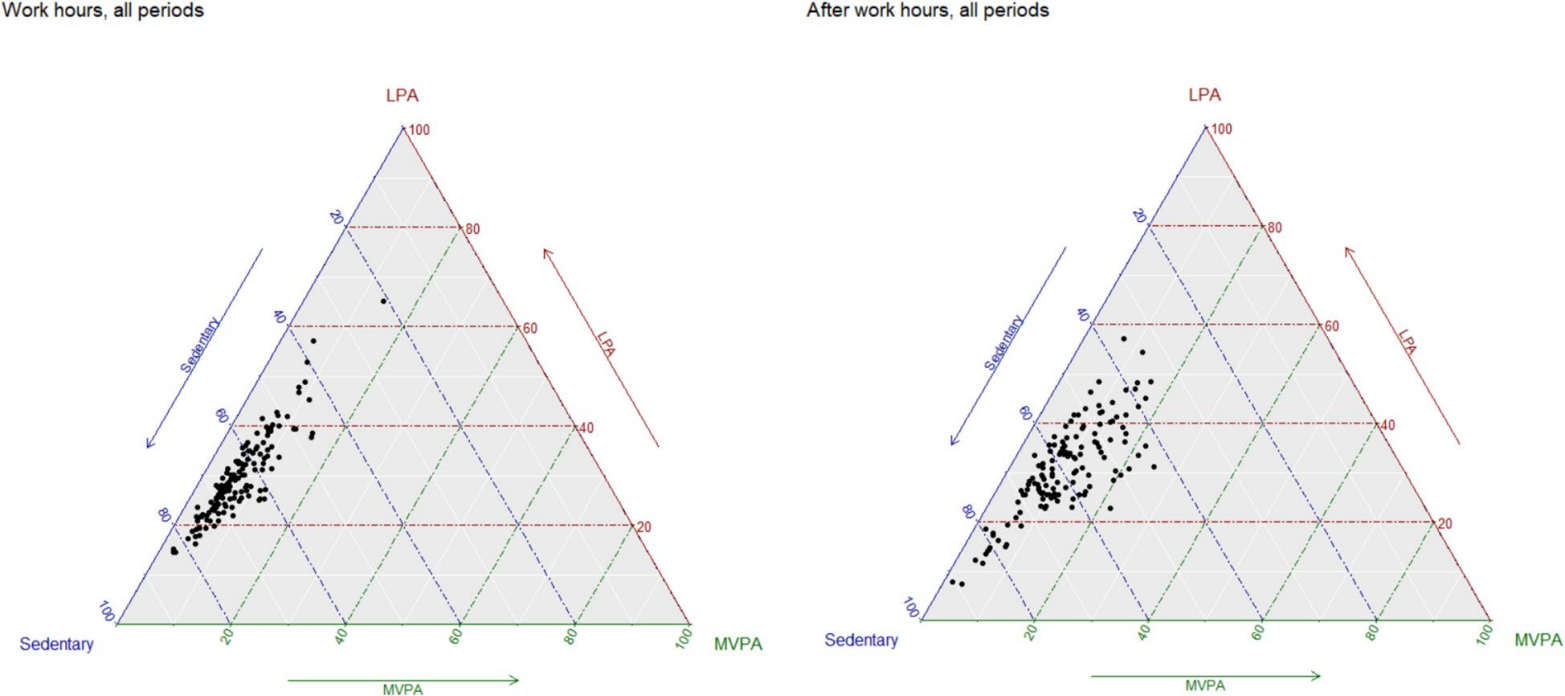
Ternary plot of the variation in time spent in sedentary, light physical activity (LPA), and moderate to vigorous physical activity (MVPA) for 63 participants during work hours (139 observations) and after work hours (135 observations)

When excluding sleep from the composition of physical behaviours, no large differences were observed between the work shift periods or between the after-work periods. However, the composition of being sedentary, LPA, and MVPA, visualised in ternary plots (Fig. 4), showed a larger variation in MVPA during after work hours compared to during work hours. This suggests that the amount of MVPA during work is consistent, but there is individual variation in the amount of MVPA performed after work hours.

In the following, the effect of work shift on the composition of physical behaviours is presented for both working hours and the subsequent after-work periods. Thus, the after-work results reflect how physical behaviours after work are influenced by the preceding shift type. These analyses are based on sub-samples; participants with work periods during all three shift types (*n =* 23), with corresponding periods after work (*n =* 20), and participants with work periods during night shifts and 24-h shifts (*n =* 34), with corresponding periods after work (*n =* 30), as described above. The descriptive patterns of physical behaviours for these sub-samples are comparable with the pattern of the whole sample. The detailed descriptive statistics are available in additional file 1, Table S1-S4.

The analyses of the subsample with all three work shifts are presented in Table 4 and, since no sleep is present during day shifts, only include a three-part composition (being sedentary, LPA, and MVPA). The repeated-measures MANOVA showed no significant overall effect of shift when shift was included as a within-subject factor and the two ILRs were used as dependent variables, either during work (F = 2.51, *p =* 0.076, η_p_^2^ = 0.35) or after work (F = 1.55, *p =* 0.236, η_p_^2^ = 0.28). Nor did the univariate tests show any statistically significant association between shift and the different ILRs.

**Table 4:** Effect of shift^*^, univariate tests, on physical behaviours expressed as isometric log ratios (ILRs)

	*F*-value (*p*-value)	Effect size, η_p_^2^
Work, *n =* 23		
ILR_1_: active^#^/sedentary	1.69 (0.196)	0.07
ILR_2_: MVPA/LPA	1.91 (0.160)	0.08
After work, *n =* 20		
ILR_1_: active^#^/sedentary	1.80 (0.179)	0.09
ILR_2_: MVPA/LPA	2.69 (0.081)	0.12

^*^Day, night, and 24-h shifts included, η_p_^2^: Partial eta squared

^#^Active = MVPA x LPA, MVPA: Moderate to vigorous physical activity, LPA: Light physical activity

The analyses of the subsample with night shifts and 24-h shifts are presented in Table 5. In addition to the analyses in Tables 4, 5 also includes sleep, i.e., a four-part composition (sleep, being sedentary, LPA and MVPA). For work, the repeated-measures MANOVA revealed a statistically significant effect of shift (F = 7.32, *p <* 0.001, η_p_^2^ = 0.42), with shift as the within-subject factor and the three ILRs as dependent variables. The univariate tests showed a statistically significant strong association of awake time relative to sleep time (ILR_3_), (η_p_^2^ = 0.34, *p <* 0.001), indicating that the overall association was largely based on the difference in the proportion of sleep during night shifts compared to 24-h shifts (32% vs 19%).

**Table 5:** Effect of shift^*^, univariate tests, on physical behaviours expressed as isometric log ratios (ILRs)

	*F*-value (*p*-value)	Effect size, η_p_^2^
Work, *n =* 34		
ILR_1_: active^#^/sedentary	0.03 (0.860)	0.00
ILR_2_: MVPA/LPA	1.29 (0.265)	0.04
ILR_3_: awake^¤^/sleep	17.34 (< 0.001)	0.34
After work, *n =* 30		
ILR_1_: active^#^/sedentary	0.58 (0.454)	0.02
ILR_2_: MVPA/LPA	4.30 (0.047)	0.13
ILR_3_: awake^¤^/sleep	0.75 (0.394)	0.03

^*^Night shifts and 24-h shifts included, η_p_^2^: Partial eta squared

^#^Active = MVPA x LPA, MVPA: Moderate to vigorous physical activity, LPA: Light physical activity

^¤^Awake = MVPA x LPA x sedentary

For after work, no overall effect of shift was observed in the repeated-measures MANOVA (F = 1.44, *p =* 0.253, η_p_^2^ = 0.14). However, the univariate tests showed a statistically significant moderate association of MVPA related to LPA (η_p_^2^ = 0.13, *p =* 0.047), indicating a larger proportion of MVPA compared to LPA after 24-h shifts compared to after night shifts (8% vs 7%).

## Discussion

This study presents new and unique findings regarding the pattern of physical behaviours among ambulance personnel and if shift types is associated to their physical behaviours. Few previous studies have used accelerometers in the EMS [30], and to the best of our knowledge, this is the first study that has used accelerometer data to describe the physical behaviours of ambulance personnel, separately for work and after work periods. We found that ambulance personnel spent 60% or more of their awake time during and after work being sedentary, and about 40% of awake time on LPA and MVPA. Type of work shift did not seem to significantly affect the pattern of physical behaviours in this group. However, when comparing only two types of shifts that also included sleep time, we found that ambulance personnel slept proportionally more during night shifts relative to their awake time compared to during the 24-h shifts.

### Physical behaviours during and after work

In the present study, we found that the ambulance personnel spent on average 95 min (during and after day shifts) to 172 min (during and after 24-h shifts) in MVPA during on average 3.6 days. Thus, these ambulance personnel exceeded the WHO general recommendations of performing at least 150 min per week in MVPA [31]. Previous studies have also found similar levels of MVPA involving personnel from the helicopter emergency service (*n =* 10) [32] and newly graduated ambulance personnel (*n =* 19) [33]. However, none of the above studies differentiated between activities during work and after work; moreover, these studies had fewer participants compared to our study. Thus, our study adds to the knowledge by presenting results from a larger sample size within this occupation and with results separately for work and after work.

The high levels of MVPA and LPA during work in the present study could reflect the high physical work demands previously described among this group based on questionnaires [7] and observations [8]. However, the ambulance personnel also had high level of MVPA and LPA after work hours unlike other occupational groups with physically demanding jobs that tend to be more sedentary and less active during non-work periods [34]. These results indicate a better sense of keeping a healthy and active lifestyle thus ensuring better physical capacity that is crucial for ensuring a safe work environment and patient care [35].

The proportion of sedentary time during work was above 60% of awake time in the present study. Given the nature of the work in the EMS, where a lot of time is spent seated in the ambulances during emergency calls and waiting for the next call at the stations, this result was expected. The WHO recommend limiting sedentary time, due to its potential negative health effects [31]. However, sitting time in the ambulances is an integral aspect of the job among ambulance personnel and thus is difficult to avoid. However, it is not known how much sedentary time is optimal in this occupational group that mitigates negative impact of prolong sedentary time and support recovery from the physical demands of the work. Therefore, further research is needed to identify the optimal balance of sedentary time and time spent in LPA and MVPA in the EMS.

### Physical behaviours related to shift type

The results of this study didn’t find an association between work shifts and physical behaviour during and after work. In the descriptive in Tables 2 and 3, there are some differences in physical behaviours between shift types. However, the association between shift type and physical behaviour turned out to be statistically non-significant. One reason could be the low sample size leading to low power in the present study. Future studies with larger sample sizes are needed to confirm the results.

Although not statistically significant in the MANOVA, the proportion of MVPA tended to be higher after night shifts and 24-h shifts compared to after day shifts. These findings are consistent with prior research on ambulance personnel, where the highest level of MVPA was observed during days including night shifts [32, 36], and the lowest level during days including day shifts [33]. One plausible explanation for these differences is that after day shifts, there are other activities that need to be prioritised, such as household responsibilities or caring for children, which could limit the time available for engaging in physical activity. While, after night shifts and 24-h shifts, individuals may have more leisure time, which could lead to increased opportunities for physical activity. Moreover, if work during night and 24-h shifts included sleep hours, less time would need to be spent on sleep after work, which could further contribute to increased available time for physical activity. Contrary findings have been reported in one study where more moderate activity was observed during days including day shifts compared to days including night shifts [37]. However, since none of these studies differentiated between work and after work hours, more research is needed to confirm our results.

Even though ambulance personnel are allowed to sleep during night and 24-h shifts, the quality of sleep during work shifts may be affected. In this study the participants’ sleep during work consisted of up to five shorter bouts of sleep, potentially disrupting the normal sleep cycle and impeding the ability to reach deeper levels of sleep [38]. Moreover, the awareness of being on call is also likely to disturb sleep quality. Results from previous studies regarding sleep among ambulance personnel found more sleep problems [39], poorer sleep quality [39, 40], and shorter sleep duration [40] compared to the general population, which might be related to shift work, but also to interrupted sleep during work hours [41]. In the present study, participants’ self-reported sleep duration aligned well with device-based sleep measured from midnight to midnight (Table 1). However, the total sleep duration was slightly shorter than that reported in a recent meta-analysis of the general population [42], which is consistent with previously identified sleep-related challenges among ambulance personnel.

When comparing two types of shifts that inherently included sleep time, night shift and 24-h shift, we found that shift was significantly associated with the composition of physical behaviours (η_p_^2^ = 0.42, *p <* 0.001). Specifically, we found that difference in the proportion of sleep compared to awake time, with a higher proportion of sleep during night shifts. Notably, both the night shifts and the 24-h shifts included an average of about 4.5 h of sleep (4.40 vs. 4.17, additional file 1, Table S1); however, due to a nearly 10-h difference in mean total hours, the composition differs. This difference is important, as working a 24-h shift with only 4.17 h of sleep should theoretically be more demanding compared to a 14-h shift with a similar duration of sleep. In addition to the risk of cognitive impairments associated with long working hours and extended waking periods [43], the 24-h shift, starting from seven in the morning, will not allow the ambulance personnel to prepare for being awake for the whole night. However, only minimal differences were observed in the composition of physical behaviours after night shifts and 24-h shifts. As one of the first studies to describe the pattern of physical behaviours among ambulance personnel and to analyse the association to different work shifts, this study adds important knowledge. However, future research is needed to determine the optimal pattern of physical behaviours during and after work for this occupational group.

### Strengths and limitations

This is one of the first studies that have used accelerometers to measure physical behaviours among ambulance personnel, and with very high compliance. Moreover, this study distinguishes between physical behaviours during work and after work. Another strength is the high wear time of accelerometers by the participants, mean 165 h or almost seven days, of which the working days were included in this study. A further strength is that the information about the accelerometers, and guidance on how to fill in the short diary was given face-to-face to each participant. During these meetings, the accelerometers were attached in the correct position and the initial reference measurement was conducted. This probably contributes to the high compliance observed in wearing the devices for seven days, adhering to the instructions regarding the diary and reference measurements, and to the fact that the data are, in principle, free from non-wear time. Moreover, the use of Acti4 is a strength since it is not based on counts threshold, but posture and movement identification.

Another strength in this study lies in its innovative approach to handling the seven-day physical behaviour data. Traditionally, days have been defined from midnight to midnight, which results in artificially splitting a continuous period that spans midnight into two separate periods. This approach is not optimal for handling data in shift workers, especially those working during nighttime hours with shifts crossing midnight hours. To address this issue, we adopted a new method where each period of the day (as defined in Fig. 2) was treated as a composition, effectively mitigating the problem of artificially dividing the period at midnight.

The data collection was conducted during the ambulance personnel’s ordinary work schedule. All measurements started with a work shift, but after that, the seven days encompassed individual variations regarding work shifts, after-work periods, and non-working days. This approach is a strength since it gives a picture of the variation in the work schedule. However, it is also a limitation since not all participants had all the work shifts and all the after-work periods during the seven days of data collection. The use of the short diary where the participants reported work or non-work time, and awake or sleep time, is a strength of this study. To further avoid possible misclassification of time, all accelerometer data were manually viewed, ensuring the accuracy of the data.

A limitation in the present study is that we did not adjust for confounders or covariates in the MANOVA, due to limited sample size. Thus, future studies should include larger sample sized to be able to adjust for variables such as age, sex, and BMI. It should be noted that ambulance personnel with an interest in physical activity, exercise, and health might have had a greater willingness to participate in the study. This potential selection bias might, at least in part, explain the high levels of MVPA and LPA during both work and after work observed in this study. Other limitations include the lack of information regarding the participants’ physical capacity, and whether they performed exercise during work shifts. Other limitations include the lack of information on participants’ physical capacity, whether they engaged in exercise during work shifts, and the number of emergency calls handled during each shift, all of which may have influenced workload and activity patterns. When interpreting these findings, it is important to consider the characteristics of the study sample. The age and gender distribution observed in our sample aligns with both previous studies [2, 32] and national workforce data for Swedish ambulance personnel [44], indicating that our sample is broadly representative for this occupational group. Furthermore, the Covid-19 pandemic during the data collection may have affected the pattern of physical behaviours, probably mainly after work due to limited opportunities to exercise in fitness centres, or to participate in group exercises.

## Conclusions

This study presents unique findings where objectively measured physical behaviours showed that ambulance personnel were physically active both during and after work. At the same time, work hours entailed a substantial amount of sedentary time. The type of shift was not associated with the pattern of physical behaviours among ambulance personnel, except concerning awake time relative to sleep when comparing night and 24-h shifts. Similar studies are needed employing large sample size to confirm the results of the study.

## Supplementary Information

Additional file 1: Method. Additional information regarding participants and data collection, questionnaire, and compositional data analysis. Descriptive statistics for cases analysed using a MANOVA. Table S1. Mean time spent in different activities during three different work shifts (*n*=23). Table S2. Mean time spent in different activities during three different after-work periods (*n*=20). Table S3. Mean time spent in different activities during night shifts and 24-hour shifts (*n*=34).Table S4. Mean time spent in different activities after night shifts and after 24-hour shifts (*n*=30). 

## Data Availability

The dataset generated and analysed during the current study are not publicly available due to privacy and ethical restrictions. Requests regarding the data can be sent to the corresponding author.

## Abbreviations

LPA: Light Physical Activity
MVPA: Moderate to Vigorous Physical Activity
EMS: Emergency Medical Services
WAI: Work Ability Index
BMI: Body Mass Index
CoDA: Compositional Data Analysis
ILR: Isometric log-ratio
WHO: World Health Organization
EU-OSHA: European Agency for Safety and Health at Work
