# Supplementary material for: Uncovering the pattern of physical behaviours among Swedish ambulance personnel: is the type of work shift important?
Source: BMC Public Health. 2026 Apr 21;26:1318. doi: 10.1186/s12889-026-27335-y (PMC13101331; doi:10.1186/s12889-026-27335-y)
Supplement: Supplementary file 1 — Additional file 1: Method. Additional information regarding participants and data collection, questionnaire, and compositional data analysis. Descriptive statistics for cases analysed using a MANOVA. Table S1. Mean time spent in different activities during three different work shifts (n=23). Table S2. Mean time spent in different activities during three different after-work periods (n=20). Table S3. Mean time spent in different activities during night shifts and 24-hour shifts (n=34).Table S4. Mean time spent in different activities after night shifts and after 24-hour shifts (n=30). [file 12889_2026_27335_MOESM1_ESM.docx]

**Supplementary materials**

**Method**

**Participants and data collection**

The first author met each participant at their workplace at one of the 10 ambulance stations, about 30 minutes before their work shift started. Instructions regarding the accelerometers were given, and the accelerometer monitoring started during the meeting. The participants wore two accelerometers, one on the thigh and one on the upper back, for seven days and nights. They were instructed to wear the accelerometers during the whole measurement period, including during showering, and to remove them only if they went swimming or if they experienced any discomfort.

A short paper-based diary was used by the participants to note the start and end of each work shift, sleep, and non-wear time. They were also instructed to note the time of the reference measures, i.e. to stand still for 15 seconds in an upright and neutral position, make a small jump, and then to stand still for another 15 seconds in an upright and neutral position. The reference measures were made once a day during the wear time, to calibrate the position of the two accelerometers.

In case of differences between the activity registered in the short diary and the accelerometer registration, e.g., concerning the time of getting up in the morning and going to bed at night, the time registered by the accelerometers was used. The times of reference measurements were registered by the participants in the short diary every day and adjusted using a specific procedure in Acti4.

**Questionnaire**

The Work Ability Index (WAI) [1] was used to create an overall index of work ability. The WAI includes seven items regarding work ability in relation to work demands, the participant's health status, sick leave, and mental resources. The index is a summary measurement of the seven items, with a total score of 7–49, and with four sub-scores: 7–27 (bad work ability), 28–36 (moderate work ability), 37–43 (good work ability), and 44–49 (very good work ability). Self-rated health was assessed using a five-point scale with the endpoints 1 = *very bad* and 5 = *very good.* The participants were asked about their average sleep length in hours, and their self-rated sleep quality on a five-point scale with the endpoints 1 = *very bad* and 5 = *very good.* Self-rated overall leisure-time physical activity level was assessed using four predefined categories (sedentary, light, moderate, and vigorous), with participants selecting the category that best reflected their activity based on descriptive examples provided for each level. Sedentary leisure time was defined as moving very little, taking only occasional walks. Light physical activity included activities such as walking or cycling at a leisurely pace, usually 1–3 hours per week. Moderate physical activity included brisk walking, cycling at a faster pace, jogging, swimming, or ball games, typically performed for around 30 minutes on most days. Vigorous physical activity encompassed running, skiing, aerobic exercise, or ball games, performed regularly for more than 3 hours per week. Although the questionnaire regarding self-rated physical activity has not been formally validated, it is used in Swedish occupational health services and has been applied in previous research [2]. The participants self-reported their weight and height, which were used to calculate their body mass index (BMI), dividing kilograms by metres squared. From the BMI, the participants were categorised into normal weight (<25), overweight (25–29.9), and obese (≥30).

**Compositional data analysis**

The isometric log ratios (ILRs) in this study, presented below, were chosen to highlight specific contrasts related to the aim of this study.

Two different sets of ILRs were used. The first was based on a composition of three parts (being sedentary, LPA, and MVPA), and was used to analyse the association of physical behaviours and shift types for participants with data from all three work shifts (n=23) and the corresponding after work periods (n=20).

ILR_1_ = $\sqrt{\frac{2}{3}}\ln\frac{\sqrt[2]{MVPA x LPA}}{sedentary}$

ILR_2_ = $\sqrt{\frac{1}{2}}\ln\frac{MVPA}{LPA}$

The second was based on a composition of four parts (sleep, being sedentary, LPA, and MVPA), and was used to analyse the association of physical behaviours and shift types for participants with data from night- and 24-hour shifts (n=34) and the corresponding after work periods (n=30).

ILR_1_ = $\sqrt{\frac{2}{3}}\ln\frac{\sqrt[2]{MVPA x LPA}}{sedentary}$

ILR_2_ = $\sqrt{\frac{1}{2}}\ln\frac{MVPA}{LPA}$

ILR_3_ = $\sqrt{\frac{3}{4}}\ln\frac{\sqrt[3]{MVPA x LPA x sedentary}}{sleep}$

**Descriptive statistics for cases analysed using a MANOVA**

**Table S1**. Mean time spent in different activities during three different work shifts (n=23).

|  |  | Day shift  (44 shifts), n=23 | | | Night shift  (27 shifts), n=23 | | | 24-hour shift  (23 shifts), n=23 | | |
| --- | --- | --- | --- | --- | --- | --- | --- | --- | --- | --- |
|  | | Mean h (SD) | % | Range | Mean h (SD) | % | Range | Mean h (SD) | % | Range |
| **Total time** | | 9.57 (1.17) |  | 7.11-11.50 | 14.58 (1.09) |  | 12.0-17.0 | 24.12 (0.30) |  | 23.98-25.0 |
|  | Sleep | 0 (0) | 0% |  | 4.05 (2.41) | 28% | 0-9.5 | 4.27 (2.25) | 18% | 0-8.0 |
|  | Sedentary | 5.98 (1.17) | 62% | 2.99-7.88 | 6.76 (1.88) | 46% | 2.20-9.90 | 12.69 (2.33) | 53% | 8.82-18.41 |
|  | LPA | 2.92 (0.88) | 31% | 1.55-4.60 | 3.15 (1.47) | 22% | 0.73-6.73 | 5.86 (1.36) | 24% | 3.79-9.67 |
|  | MVPA | 0.67 (0.25) | 7% | 0.30-1.26 | 0.62 (0.28) | 4% | 0.15-1.21 | 1.30 (0.43) | 5% | 0.66-2.33 |

LPA: Light physical activity, MVPA: Moderate to vigorous physical activity, h: hours, SD: Standard Deviation

**Table S2**. Mean time spent in different activities during three different after-work periods (n=20).

|  |  | After day shift  (36 periods), n=20 | | | After night shift  (22 periods), n=20 | | | After 24-hour shift  (20 periods), n=20 | | |
| --- | --- | --- | --- | --- | --- | --- | --- | --- | --- | --- |
|  | | Mean h (SD) | % | Range | Mean h (SD) | % | Range | Mean h (SD) | % | Range |
| **Total time** | | 14.75 (4.85) |  | 6.68-23.50 | 21.97 (4.62) |  | 15.0-32.50 | 18.50 (4.85) |  | 9.00-24.50 |
|  | Sleep | 6.69 (2.54) | 45% | 2.00-12.50 | 6.69 (3.24) | 29% | 0.08-11.08 | 4.81 (3.15) | 25% | 0.0-10.17 |
|  | Sedentary | 4.41 (1.53) | 31% | 2.03-7.30 | 9.20 (2.64) | 43% | 5.72-15.89 | 7.94 (1.96) | 44% | 3.60-11.22 |
|  | LPA | 2.74 (1.45) | 18% | 0.69-5.97 | 4.77 (1.74) | 22% | 1.29-7.88 | 4.33 (1.79) | 23% | 1.22-7.24 |
|  | MVPA | 0.89 (0.63) | 6% | 0.22-2.66 | 1.31 (0.74) | 6% | 0.25-2.85 | 1.42 (0.62) | 8% | 0.53-2.43 |

LPA: Light physical activity, MVPA: Moderate to vigorous physical activity, h: hours, SD: Standard Deviation

**Table S3**. Mean time spent in different activities during night shifts and 24-hour shifts (n=34).

|  |  | Night shift  (47 shifts), n=34 | | | 24-hour shift  (35 shifts), n=34 | | |
| --- | --- | --- | --- | --- | --- | --- | --- |
|  | | Mean h (SD) | % | Range | Mean h (SD) | % | Range |
| **Total time** | | 14.57 (1.05) |  | 12.0-17.0 | 24.11 (0.38) |  | 23.08-25.0 |
|  | Sleep | 4.40 (2.40) | 32% | 0-9.5 | 4.17 (2.19) | 19% | 0-8.0 |
|  | Sedentary | 6.49 (2.00) | 43% | 2.20-11.67 | 12.94 (2.06) | 53% | 8.82-18.41 |
|  | LPA | 3.04 (1.25) | 21% | 0.73-6.73 | 5.70 (1.46) | 23% | 3.23-9.67 |
|  | MVPA | 0.64 (0.25) | 4% | 0.15-1.21 | 1.30 (0.44) | 5% | 0.66-2.33 |

LPA: Light physical activity, MVPA: Moderate to vigorous physical activity, h: hours, SD: Standard Deviation

**Table S4**. Mean time spent in different activities after night shifts and after 24-hour shifts (n=30).

|  |  | After night shift  (40 periods), n=30 | | | After 24-hour shift  (31 periods), n=30 | | |
| --- | --- | --- | --- | --- | --- | --- | --- |
|  | | Mean h (SD) | % | Range | Mean h (SD) | % | Range |
| **Total time** | | 21.70 (4.27) |  | 15.00-32.50 | 20.09 (7.71) |  | 7.50-34.00 |
|  | Sleep | 6.25 (3.25) | 28% | 0.0-11.08 | 5.37 (3.73) | 26% | 0.0-14.00 |
|  | Sedentary | 9.19 (2.40) | 43% | 5.72-15.89 | 8.56 (3.02) | 44% | 3.36-17.61 |
|  | LPA | 4.78 (1.72) | 22% | 1.29-8.20 | 4.56 (2.12) | 22% | 0.56-7.97 |
|  | MVPA | 1.48 (0.86) | 7% | 0.25-3.37 | 1.60 (1.01) | 8% | 0.26-5.01 |

LPA: Light physical activity, MVPA: Moderate to vigorous physical activity, h: hours, SD: Standard Deviation

**References**

1. Ilmarinen J: **The Work Ability Index (WAI)**. *Occup Med* 2007, **57**(2):160.

2. Johnsen AM, Wagman P, Broström A, Fransson EI: **Work-, lifestyle-, and health-related factors among women and men working in the emergency medical services**. *Int J Occup Saf Ergon* 2024, **30**(2):651–661.
